# Supplementary material for: Constitutive activation of DIA1 (DIAPH1) via C‐terminal truncation causes human sensorineural hearing loss
Source: EMBO Mol Med. 2016 Oct 5;8(11):1310–24. doi: 10.15252/emmm.201606609 (PMC5090661; doi:10.15252/emmm.201606609)
Supplement: Supplementary file 1 — Expanded View Figures PDF [file EMMM-8-1310-s001.pdf]

Expanded View Figures

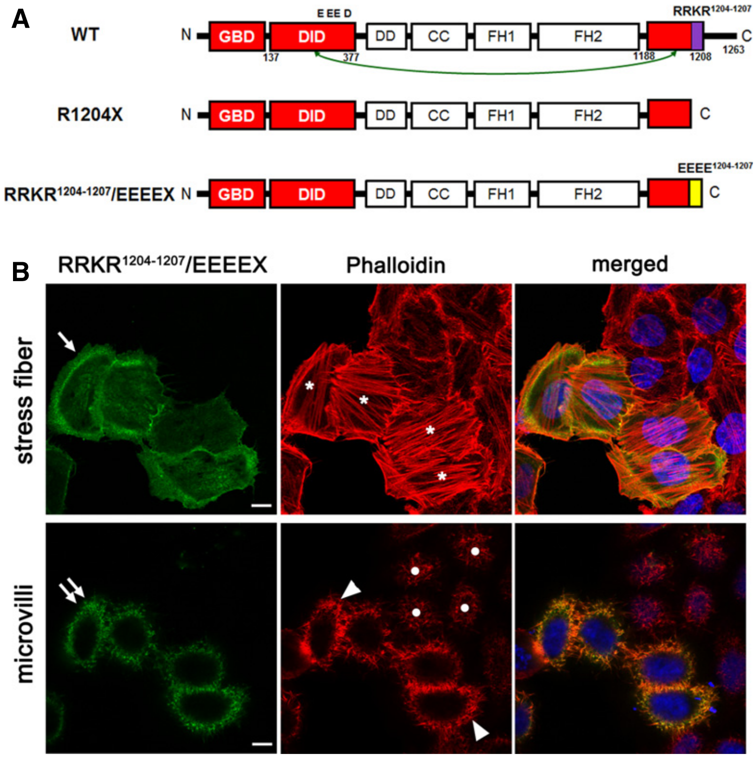

Figure EV1. DIA1(RRKR$^{1204–1207}$/EEEEX) induces elongated microvilli and enhances stress fiber formation in HeLa cells.

A Illustrations of WT DIA1 (contains four basic amino acids at the DAD C-terminus), and the R1204X (basic amino acids absent) and RRKR$^{1204–1207}$/EEEEX (basic-to-acidic amino acid swaps) mutants.

B GFP-tagged DIA1(RRKR$^{1204–1207}$/EEEEX) was transfected into HeLa cells and fixed 24 h after transfection. Fixed cells were stained with Alexa568-conjugated phalloidin and DAPI (blue) and observed under a confocal laser microscope. Note the localization of GFP-DIA1(RRKR$^{1204–1207}$/EEEEX) to the plasma membrane (arrow) and microvilli (double arrows) and induction of enhanced stress fiber (asterisks) and elongated/thick microvillus formation (arrowheads) by GFP-DIA1(RRKR$^{1204–1207}$/EEEEX) expression, compared with that in nonexpressing cells (circles). Scale bars: 10 μm. 3D movie is available in Movie EV4. Representative of five experiments.

 © 2016 The Authors

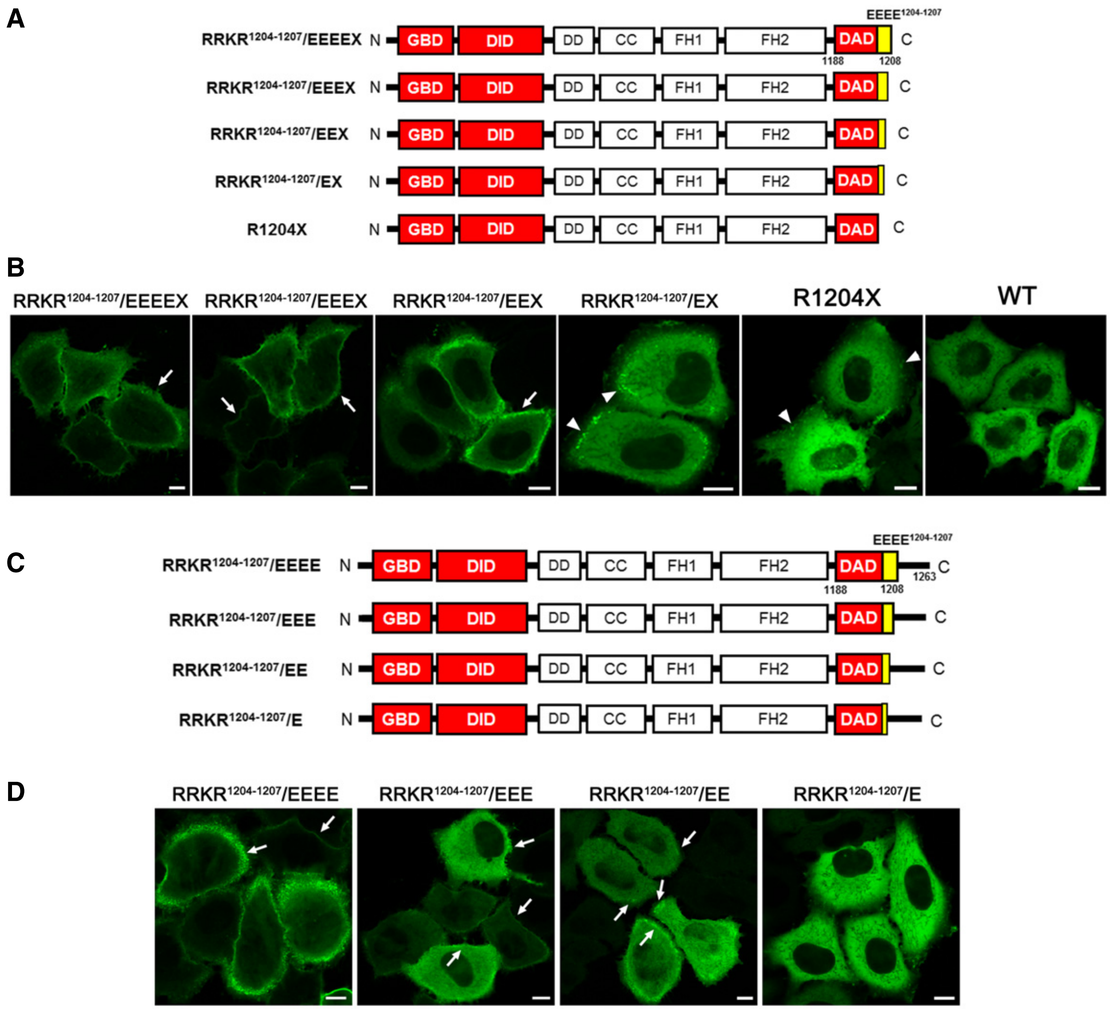

Figure EV2. R1204X mutation in the DAD generates a mildly active DIA1, and the C-terminal aa (56 aa) after the DAD is a negative regulator of the intramolecular DID-DAD interaction.

A Illustrations of the C-terminal truncation DIA1 mutants with 0–4 acidic amino acids (aa) at the DAD C-terminus.

B GFP-tagged WT DIA1 and mutants containing 0–4 acidic aa substitutions were transfected into HeLa cells. Twenty-four hours after transfection, plasma membrane (PM) localization was observed using a confocal laser microscope. The degree of PM localization (arrows) is shown from left to right: $RRKR^{1204-1207}$/EEEEX (strongest) and WT (no PM localization). Note dot-like localization in areas adjacent to the PM (arrowheads) in GFP-$RRKR^{1204-1207}$/EX- and GFP-R1204X-expressing cells. Scale bars: 10 μm. Representative of five experiments.

C Illustrations of DIA1 mutants with mutation of the acidic amino acids in the $RRKR^{1204-1207}$ motif at the DAD C-terminus.

D GFP-tagged $RRKR^{1204-1207}$/E, $RRKR^{1204-1207}$/EE, $RRKR^{1204-1207}$/EEE, and $RRKR^{1204-1207}$/EEEE mutants were transfected into HeLa cells. Twenty-four hours after transfection, plasma membrane (PM) localization was observed using a confocal laser microscope. Increasing PM localization of these mutants (arrows): $RRKR^{1204-1207}$/EE < $RRKR^{1204-1207}$/EEE < $RRKR^{1204-1207}$/EEEE, was observed. Note no apparent (or dot-like) PM localization was observed with GFP-$RRKR^{1204-1207}$/E, and that the dot-like localization of $RRKR^{1,204-1,207}$/E was significantly weaker than that of $RRKR^{1204-1207}$/EX (see B). Scale bars: 10 μm. Representative of five experiments.

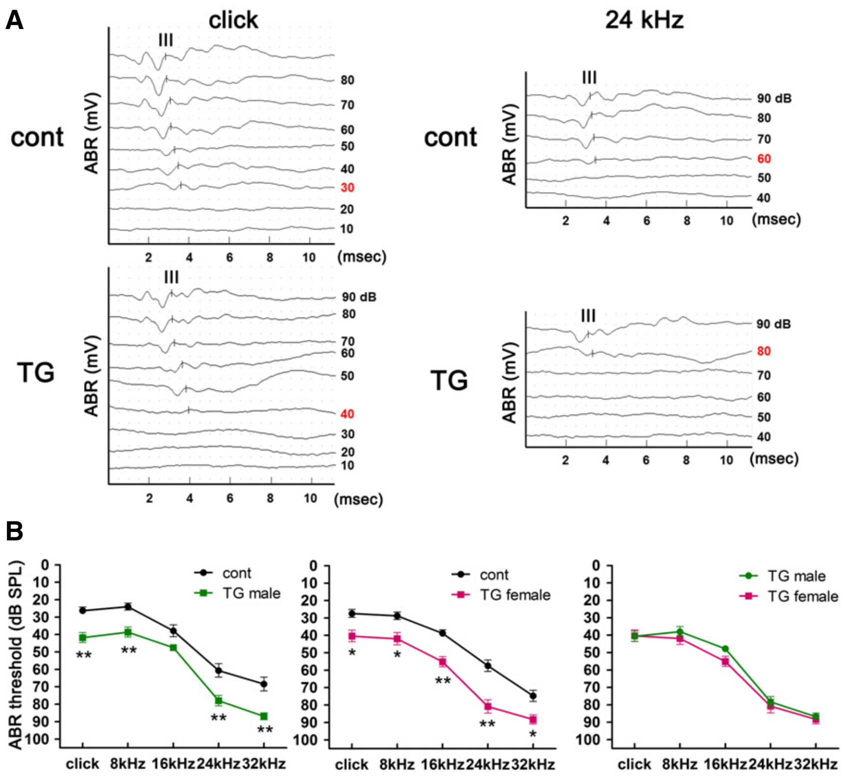

Figure EV3. Representative traces of ABR from control and TG mice, and no sex-based difference in ABR of DIA1(R1204X)-TG mice.

A Example traces of click (2–4 kHz) and 24 kHz ABR from the two genotypes (control and DIA1 (R1204X)-TG mice) at 25 weeks of age. ABR thresholds are indicated in red: 30 dB SPL and 60 dB SPL in control mice and 40 dB SPL and 80 dB SPL in TG mice for click and 24 kHz stimulations, respectively.

B Click and pure tone-burst (8, 16, 24, 32 kHz) ABR thresholds (dB SPL, mean ± SE) in male (control: n = 18, DIA1(R1204X)-TG: n = 24) and female (control: n = 15, DIA1(R1204X)-TG: n = 18) mice at 25 weeks of age. $^{**}P$ = 0.0007 (click), $^{**}P$ = 0.0017 (8 kHz), $^{**}P$ = 0.0001 (24 kHz), and $^{**}P < 0.0001$ (32 kHz) in male mice and $^{*}P$ = 0.0160 (click), $^{*}P$ = 0.0135 (8 kHz), $^{**}P$ = 0.0010 (16 kHz), $^{**}P < 0.0001$ (24 kHz), and $^{*}P$ = 0.0101 (32 kHz) by Bonferroni's *post hoc* test following two-way ANOVA in female mice. Note: no significant differences are observed between male and female TG mice.

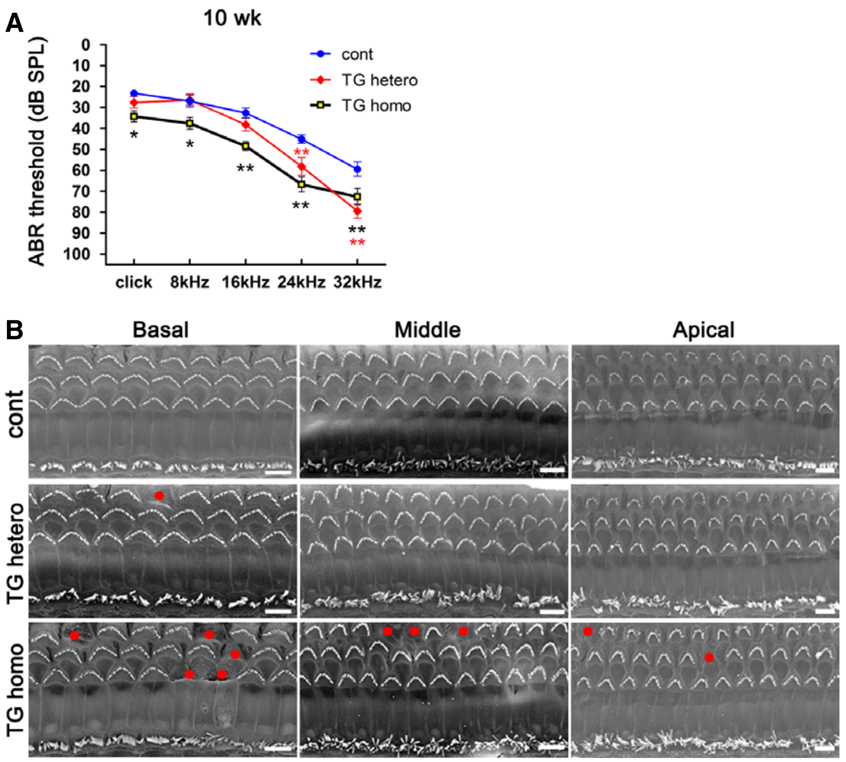

Figure EV4. Exacerbated deafness and HC loss in homozygous DIA1(R1204X)-TG mice.

A Click and pure tone-burst (8, 16, 24, 32 kHz) ABR thresholds (dB SPL, mean ± SE) in control mice (n = 16), heterozygous (n = 16), and homozygous DIA1(R1204X)-TG mice (n = 12) at 10 weeks of age. Note the significant differences at 24 kHz and 32 kHz in heterozygous TG mice and click–32 kHz in homozygous TG mice compared with control mice. $^{**}P$ = 0.0039 (24 kHz) and $^{**}P < 0.0001$ (32 kHz) in heterozygous TG mice and $^{*}P$ = 0.0352 (click), $^{*}P$ = 0.0458 (8 kHz), $^{**}P$ = 0.0010 (16 kHz), $^{**}P < 0.0001$ (24 kHz), $^{**}P$ = 0.0085 (32 kHz) in homozygous TG mice by Bonferroni's *post hoc* test following two-way ANOVA.

B The organ of Corti (OC) was dissected from 10-week-old control, and heterozygous and homozygous DIA1(R1204X)-TG mice and fixed. Prepared samples were observed under a scanning electron microscope. Exacerbated OHC loss is observed at basal, middle, and apical turns of homozygous DIA1(R1204X)-TG cochlea compared with control and heterozygous cochlea. Red circles show OHC loss. The image of the basal turn of control mice is duplicated from Fig 8B. Scale bars: 5 μm. Representative of four experiments.

 © 2016 The Authors

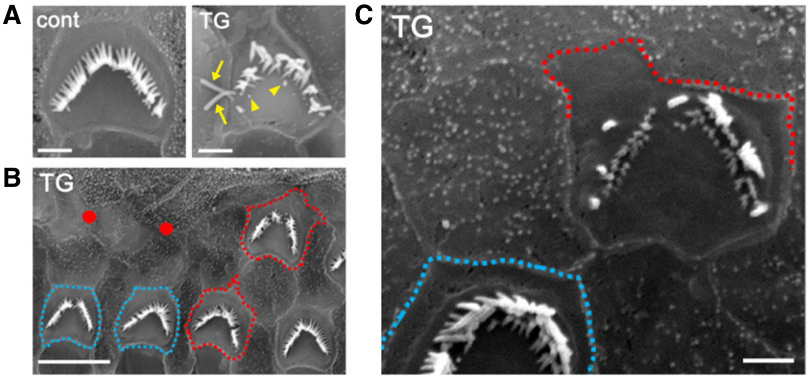

Figure EV5. Deformed cell shape in OHCs of DIA1(R1204X)-TG mice.

The organ of Corti (OC) was dissected from 25-week-old control and homozygous DIA1(R1204X)-TG mice (A, B; $n = 6$) and 42-week-old homozygous DIA1 (R1204X)-TG mice (C; $n = 2$), and fixed. Prepared samples were observed under a scanning electron microscope. OHCs with deformed or normal shapes are surrounded by dashed red lines and dashed blue lines, respectively (B, C).

- A OHC in DIA1(R1204X)-TG OC had elongated (arrows), sparse, and short (arrowheads) stereocilia. Scale bars: 1 µm.
- B Abnormal (sparse and short) stereocilia in one of the deformed OHCs (upper OHC). The spaces formed by HC loss (red circles) are replaced by other cells. Scale bar: 5 µm.
- C Deformed, but not normal OHC, have abnormal (sparse, short, and dislocated) stereocilia. Scale bar: 1 µm.

© 2016 The Authors
