# Supplementary material for: Constitutive activation of DIA1 (DIAPH1) via C‐terminal truncation causes human sensorineural hearing loss
Source: EMBO Mol Med. 2016 Oct 5;8(11):1310–24. doi: 10.15252/emmm.201606609 (PMC5090661; doi:10.15252/emmm.201606609)
Supplement: Supplementary file 2 — Table EV1 [file EMMM-8-1310-s002.zip › Table_EV1.docx]

**Table EV1**

**Summary of primer pairs used for genotyping and qPCR.**

**Primer pairs used for genotyping**

|  |
| --- |

**CAG**:

5′-TAATCAATTACGGGGTCATTAGTTCATAGC-3′

5′-TCCCATAAGGTCATGTACTGGGCATAATGC-3′

**CAG/FLAG**:

5′-CTGACTGACCGCGTTACTCCCACAG-3′ (in the CAG promoter)

5′-ATAATCACCGTCATGGTCTTTGTAGTCC-3′ (in the FLAG tag)

**CAG/DIA1-1**: described in the main text

**DIA1-2**:

5′-GGATGATCCCACAGCACAGTCATTGCAA-3′ (201–228)

5′-GACACCTCTGCTTCCAGGTCCTGTTTCT-3′ (1571–1598)

**DIA1-3**:

5′-CATCCTGCAGCACTTACTCTTGGTCCGA-3′ (1209–1236)

5′-TCTCTTGATAGGGCATGCGGAAGGAACC-3′ (2566–2593)

**DIA1-4**:

5′-TATGCCTCCACCTCCCCCATTTGGATT T-3′ (2211–2238)

5′-TGACTGCAGGGCTTCTAGAAGACTGTCC-3′ (3570–3597)

**DIA1/polyA**:

5′-ACGTGATGTTCAGAATTTCCCAGCTGCC-3′ (3138–3210)

5′-GACAAACCACAACTAGAATGCAGTGA-3′ (in the SV40 polyA)

**Primer pairs used for qPCR**

|  |
| --- |

**mouse Dia1:**

5′-CAGAATTAACAGCGCGGCAC-3′ (1469–1488)

5′-AGGCATCCTTTTCTCCTTGAAGAT-3′ (1544–1567)

**human DIA1:**

5′-GGAGTTACGATAGCCGGAACA-3′ (593–613)

5′-CTTCTGTCTCCAACATGGTCTTG-3′ (666–688)

**mouse Gapdh:**

5′-TGCACCACCAACTGCTTAGC-3′ (526–545)

5′-GGATGCAGGGATGATGTTCT-3′ (683–702)
