# Supplementary material for: Constitutive activation of DIA1 (DIAPH1) via C‐terminal truncation causes human sensorineural hearing loss
Source: EMBO Mol Med. 2016 Oct 5;8(11):1310–24. doi: 10.15252/emmm.201606609 (PMC5090661; doi:10.15252/emmm.201606609)
Supplement: Supplementary file 11 — Source Data for Figure 4B [file EMMM-8-1310-s010.pptx]

## Slide 1
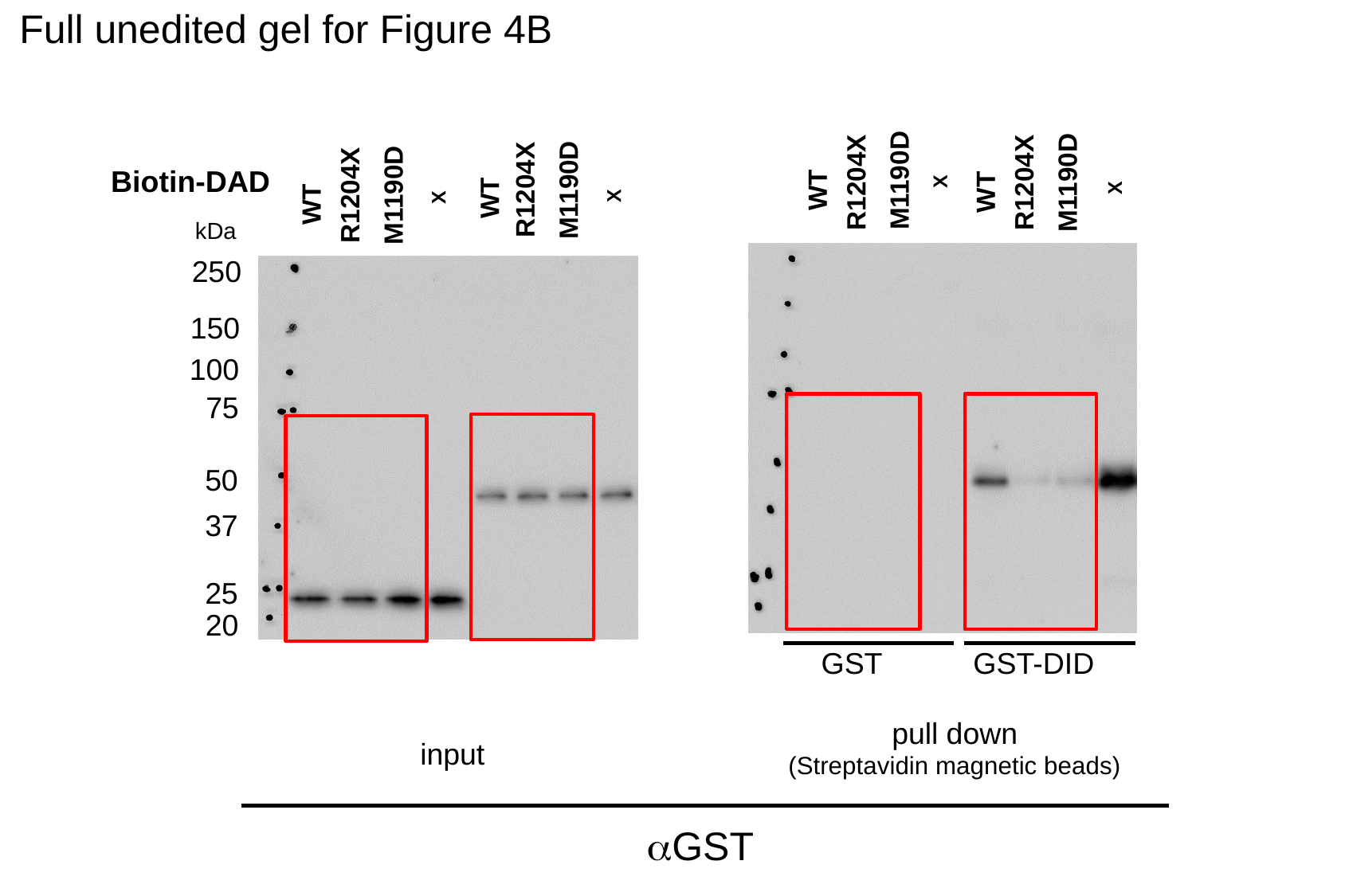

Full unedited gel for Figure 4B
Biotin-DAD
M1190D
R1204X
R1204X
M1190D
X
WT
R1204X
M1190D
WT
X
R1204X
M1190D
WT
X
X
WT
kDa
250
150
100
75
50
37
25
20
GST
GST-DID
pull down
(Streptavidin magnetic beads)
input
aGST
