# Supplementary material for: Constitutive activation of DIA1 (DIAPH1) via C‐terminal truncation causes human sensorineural hearing loss
Source: EMBO Mol Med. 2016 Oct 5;8(11):1310–24. doi: 10.15252/emmm.201606609 (PMC5090661; doi:10.15252/emmm.201606609)
Supplement: Supplementary file 12 — Source Data for Figure 7 [file EMMM-8-1310-s011.pptx]

## Slide 1
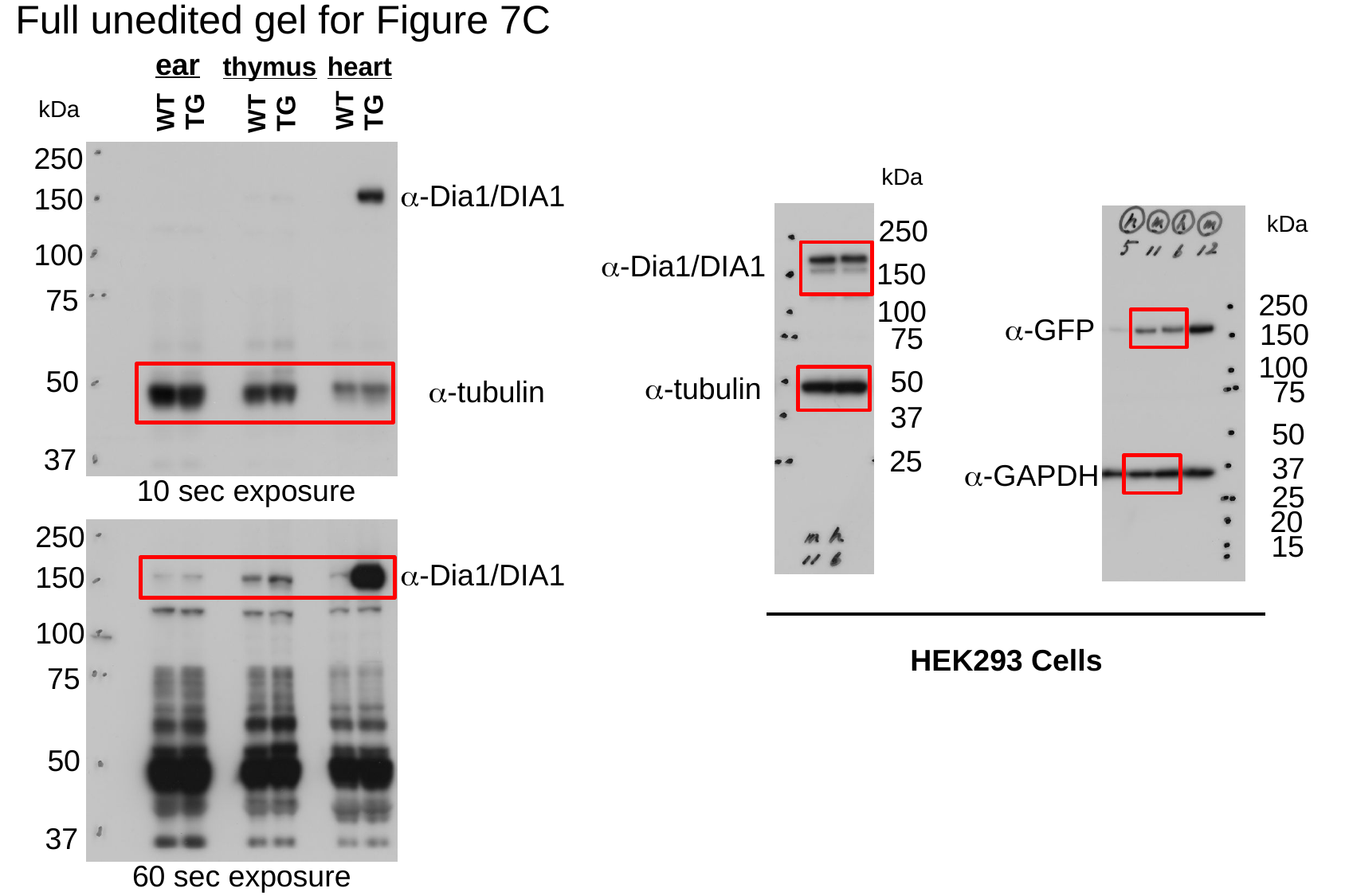

Full unedited gel for Figure 7C
ear
thymus
heart
kDa
WT
TG
TG
WT
TG
WT
250
kDa
a-Dia1/DIA1
150
kDa
250
100
a-Dia1/DIA1
150
75
250
100
a-GFP
150
75
100
50
50
a-tubulin
75
a-tubulin
37
50
37
25
37
a-GAPDH
10 sec exposure
25
20
250
15
a-Dia1/DIA1
150
100
HEK293 Cells
75
50
37
60 sec exposure

## Slide 2
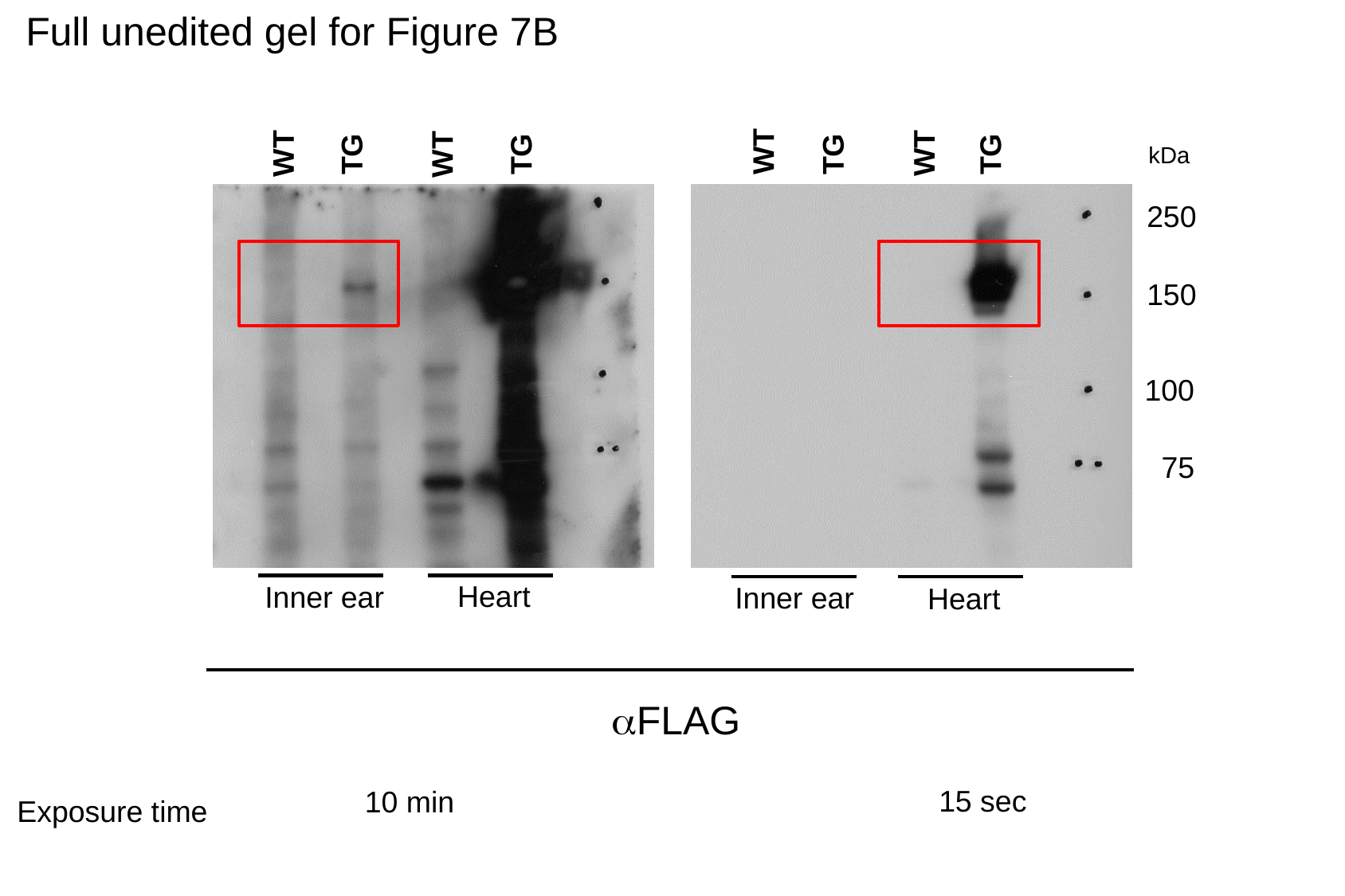

Full unedited gel for Figure 7B
WT
WT
WT
TG
TG
TG
WT
TG
kDa
250
150
100
75
Heart
Inner ear
Inner ear
Heart
aFLAG
15 sec
10 min
Exposure time
